# Supplementary material for: Transcriptomic Insights into Mechanisms of Early Seed Maturation in the Garden Pea (Pisum sativum L.)
Source: Cells. 2020 Mar 23;9(3):779. doi: 10.3390/cells9030779 (PMC7140803; doi:10.3390/cells9030779)

Zhongwan−6(10) vs Zh

Global Summary

%DE = 0.89

genes with fdr < 0.2 = 532 (313 + / 219 −)

genes with fdr < 0.1 = 522 (311 + / 211 −)

genes with fdr < 0.05 = 466 (284 + / 182 −)

genes with fdr < 0.01 = 364 (232 + / 132 −)

genes in genesets = 0

<FC> = 0

<t−score> = −3158.62

<p−value> = 0

<fdr> = 0.11

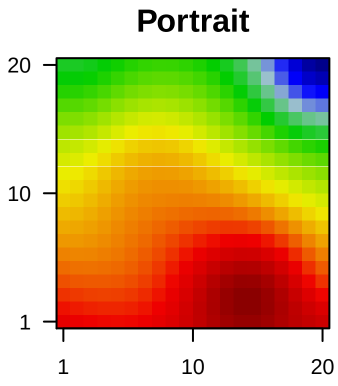

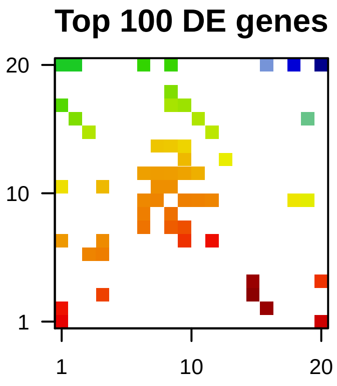

Global Genelist

Rank	ID	log(FC)	p−value	fdr	Metagene	Description
1	TRINITY_DN7	−0.44	0e+00	0.0000000[illegible]	[illegible] x 20	
2	TRINITY_DN6	−1.18	0e+00	0.0000000[illegible]	[illegible] x 20	
3	TRINITY_DN1	−0.05	4e−10	0.0000000[illegible]	[illegible] x 20	
4	TRINITY_DN4	−2.14	4e−10	0.0000000[illegible]	[illegible] x 20	
5	TRINITY_DN5	−0.54	8e−10	0.0000000[illegible]	[illegible] x 20	
6	TRINITY_DN5	−1.15	2e−09	0.0000000[illegible]	[illegible] x 20	
7	TRINITY_DN5	−0.56	1e−08	0.000003[illegible]	[illegible] x 20	
8	TRINITY_DN4	1.62	6e−08	0.000006[illegible]	[illegible] x 3	
9	TRINITY_DN1	−0.01	2e−07	0.000008[illegible]	[illegible] x 12	
10	TRINITY_DN3	−0.13	3e−07	0.000008[illegible]	[illegible] x 16	
11	TRINITY_DN4	−0.27	5e−07	0.000008[illegible]	[illegible] x 4	
12	TRINITY_DN5	1.94	5e−07	0.00001[illegible]	[illegible] x 1	
13	comp40971_c	1.1	1e−06	0.00001[illegible]	[illegible] x 2	
14	TRINITY_DN1	0.02	2e−06	0.00001[illegible]	[illegible] x 9	
15	TRINITY_DN8	−0.26	2e−06	0.00001[illegible]	[illegible] x 17	
16	TRINITY_DN4	−0.12	2e−06	0.00001[illegible]	[illegible] x 17	
17	TRINITY_DN1	0	2e−06	0.00001[illegible]	[illegible] x 12	
18	comp48352_c	−0.05	3e−06	0.00001[illegible]	[illegible] x 14	
19	TRINITY_DN3	−0.11	3e−06	0.00001[illegible]	[illegible] x 20	
20	TRINITY_DN4	−0.66	3e−06	0.00001[illegible]	[illegible] x 16	

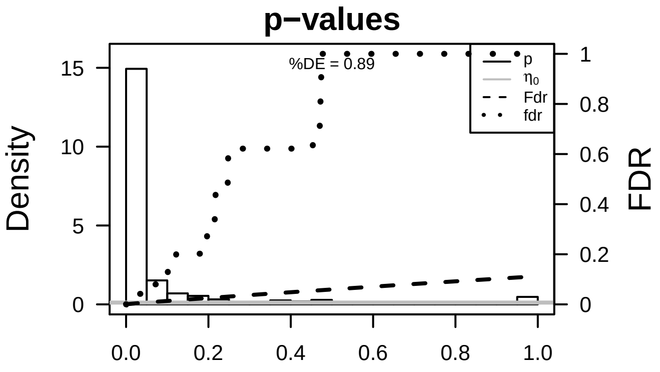

Supplement: Supplementary file 1 [file cells-09-00779-s001.zip › Supplementary materials/FileS3.zip!/FileS2/Hormones - Results/Summary Sheets - Differences/Reports/Zhongwan.6.10..vs.Zhongwan.6.25..pdf]
